# Investigation of the optimal collagen fibre orientation in human iliac arteries

**DOI:** 10.1016/j.jmbbm.2015.06.011

**Published:** 2015-06-20

**Authors:** Nan Qi, Hao Gao, Raymond W. Ogden, Nicholas A. Hill, Gerhard A. Holzapfel, Hai-Chao Han, Xiaoyu Luo

**Affiliations:** a School of Mathematics and Statistics, University of Glasgow, 15 University Gardens, Glasgow G12 8QW, UK; b Institute of Biomechanics, Graz University of Technology, Kronesgasse 5-I, 8010 Graz, Austria; c Department of Mechanical Engineering, The University of Texas at San Antonio, San Antonio, TX 78249, USA

**Keywords:** Iliac bifurcation, Constitutive law, Collagen fibre, Fibre orientation, Remodelling, Pre-stretch, Residual stress, Total energy

## Abstract

The distribution of collagen fibres plays a significant role in the mechanical behaviour of artery walls. Experimental data show that in most artery wall layers there are two (or more) in-plane symmetrically disposed families of fibres. However, a recent investigation revealed that some artery wall layers have only one preferred fibre direction, notably in the medial layer of human common iliac arteries. This paper aims to provide a possible explanation for this intriguing phenomenon. An invariant-based constitutive model is utilized to characterize the mechanical behaviour of tissues. We then use three different hypotheses to determine the ‘optimal fibre angle’ in an iliac artery model. All three hypotheses lead to the same result that the optimal fibre angle in the medial layer of the iliac artery is close to the circumferential direction. The axial pre-stretch, in particular, is found to play an essential role in determining the optimal fibre angle.

## 1. Introduction

The collagen fibres within artery walls play a central role in the macroscopic mechanical behaviour of walls (Holzapfel et al., 2000; Gasser et al., 2006). Human common iliac arteries are of particular clinical interest as atherosclerosis-prone vessels, since they frequently undergo endovascular treatment. Iliac arteries are relatively easy to access for vascular diagnostic procedures (Schulze-Bauer et al., 2003).

Artery walls are usually composed of three distinct layers, the intima, the media and the adventitia, and it is widely accepted that variations exist in both the structural composition and the material properties of artery walls in different regions of the arterial tree, even from the same individual (Mangell et al., 1996; Holzapfel et al., 2000, 2004). Collagen fibres are key ingredients in the structure of artery walls. In most of the regions, load bearing layers such as the media and the adventitia are shown to have two (or more) in-plane symmetrically disposed families of fibres.

Continuum constitutive models of arterial layers integrate information about the tissue morphology and therefore allow investigation of the interrelation between structure and function in response to mechanical loading. Carefully constructed constitutive laws based on experiments are of critical importance for analysing the physiological and pathological load-carrying mechanisms in soft tissues (Gasser et al., 2006).

A number of experimental studies, based on polarised light microscopy of stained arterial tissue, have shed light into the detailed structural organisation of the complex three-dimensional elastin, collagen and smooth muscle arrangement within the artery wall (Canham et al., 1989; Finlay et al., 1995, 1998; Schriefl et al., 2012). These studies show not only that artery tissues are highly anisotropic, but also provide an explanation for the exponential behaviour of the tissues. The gradual recruitment of the collagen fibres, which are considered as crimped in the natural configuration, is responsible for the exponentially increased stiffness of the tissue when stretched (Roach and Burton, 1957; Lanir, 1983).

With this understanding, more advanced constitutive laws have been developed to make use of the microscopic information of artery wall structures (Holzapfel et al., 2000, 2015; Gasser et al., 2006). The anisotropic elastic energy functions proposed by Holzapfel et al. (2000) and Holzapfel and Gasser (2001) represent one category of such structure-based approaches. For example, the constitutive model of Holzapfel et al. (2000) is used to represent the ground matrix and the distinct fibre families in the artery wall. The model assumes that the fibres are symmetrically disposed relative to the axial direction and have no component in the radial direction. However, this constitutive model does not include the fibre dispersion observed in experiments, and to take account of fibre dispersions, two extended models have been developed subsequently. One of these is the *ρ* model (Holzapfel et al., 2005a,b) in which a constant scalar *ρ* is introduced to account for the fibre dispersion. The other is the *κ* model which is derived from a generalised structure tensor (Gasser et al., 2006). Both the *ρ* and the *κ* models are invariant based and include the effect of fibre dispersion, but unlike *ρ*, *κ* can be directly estimated from the measured fibre density distributions using, for example, polarised light microscopy (Canham et al., 1989; Finlay et al., 1995, 1998; Schriefl et al., 2012).

Most of the aforementioned studies focussed on the mechanical properties of coronary arteries (Holzapfel et al., 2005a). In this study we concentrate on the human iliac artery. This is because an exception to the fibre structure has been found in the medial layer of human common iliac arteries in the recent work by Schriefl et al. (2012). Using polarised light microscopy on stained arterial tissues, these researchers measured the layer-specific collagen fibre density distribution in human thoracic and abdominal aortas, and in common iliac arteries. They found that unlike in most of the investigated arterial layers, where there are two or more distinct families of the collagen fibres, fibres are found to be mostly parallel to the circumferential direction in the media of the human common iliac arteries. Various fibre dispersions in different layers of arteries were also reported.

The work of Schriefl et al. (2012) raises interesting questions. In particular, what determines the optimal fibre orientation? Can we explain the fibre distribution in the media of the common iliac artery from a mechanics standpoint? In this paper, we attempt to answer these questions using a combined analytical and computational approach.

We model the iliac artery using a two-layer thick-walled model, including only the media and adventitia. We use the *κ* model in which the effect of the fibre dispersion is taken into account. Both the axial pre-stretch and circumferential residual stress are considered. To separate the effects of the circumferential residual stress and axial pre-stress from the geometric influences, we also investigate a straight tube model with the corresponding material properties as well as the residual stress in the circumferential direction. Inflation and extension experiments are simulated numerically with a mean pressure loading at 100 mmHg, since it is the mean blood pressure that is primarily regulated physiologically (Burchell, 1968; Yu et al., 1992). For simplicity, we confine our study to static loading only. Finally, three different hypotheses are used to determine the ‘optimal fibre angle’ in the iliac artery model. Results from all three hypotheses support the experimental observation that there is probably a single fibre family in the media of human iliac arteries.

## 2. Methodology

This section consists of three parts: the geometric construction of the aorto-iliac bifurcation, the determination of the material parameters in the strain–energy function, and the finite element analysis of the iliac artery model.

### 2.1. Geometry of a 3D aorto-iliac bifurcation

**Fig. 1:**
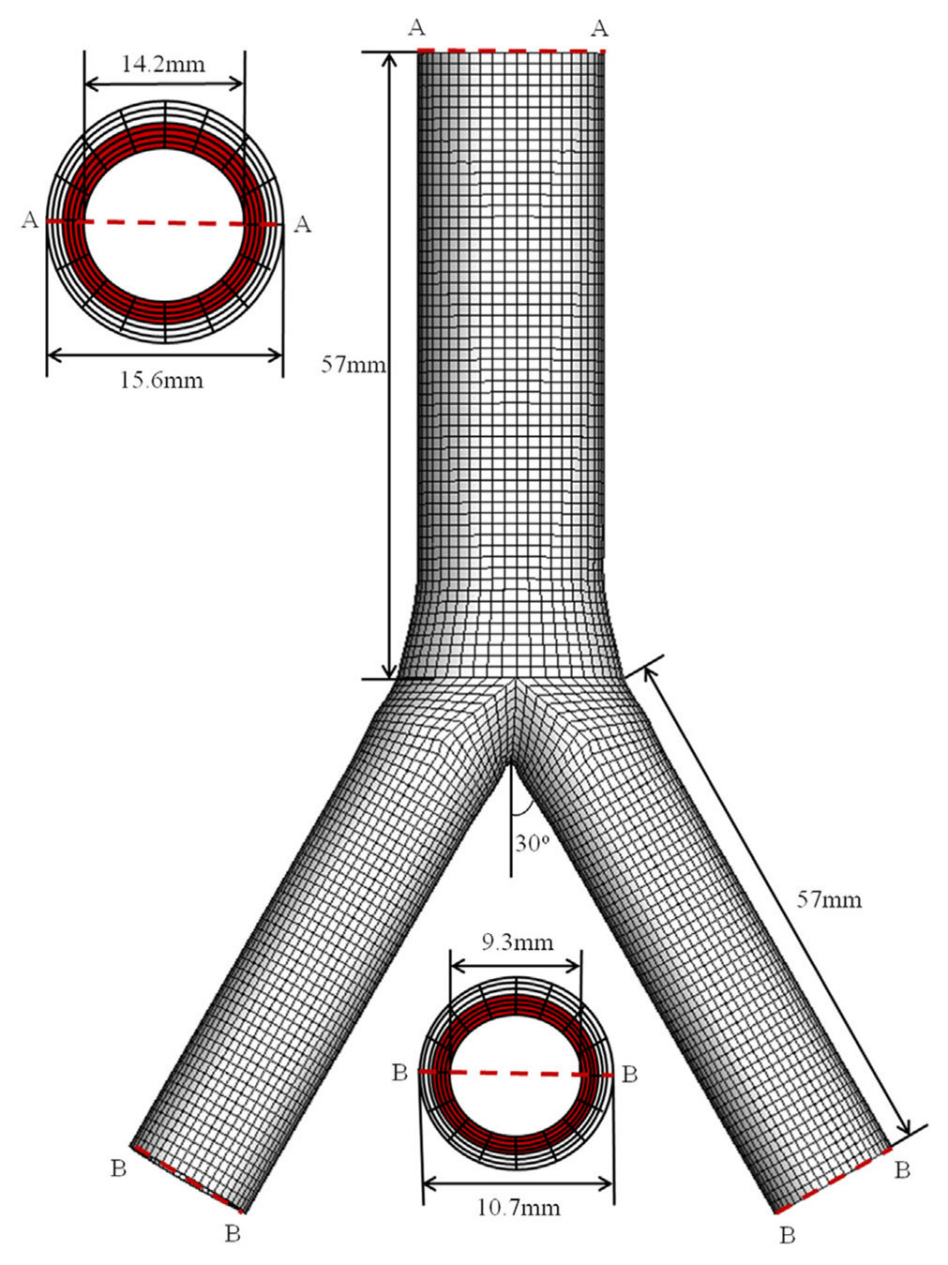
3D geometry of the aorto-iliac bifurcation model. The geometric information is taken from the literature. The inner and outer diameters are chosen to be 14.2 mm and 15.6 mm, respectively, for the abdominal aorta, and 9.3 mm and 10.7 mm, respectively, for the common iliac arteries, following Schulze-Bauer et al. (2003) and Kahraman et al. (2006). The length of the aorta and each iliac artery is taken to be 57 mm (Stergiopulos et al., 1992; Olufsen, 1998). The two iliac branches are assumed to deviate from the centreline of the aorta symmetrically at 30° (Shah et al., 1976; Long et al., 2000). The axial lengths of the elliptical transition region approaching from the aorta and the iliac branches are 9 mm and 15 mm, respectively. (For interpretation of the references to colour in this figure caption, the reader is referred to the web version of this paper.)

Based on human data documented in the literature (Stergiopulos et al., 1992; Olufsen, 1998; Schulze-Bauer et al., 2003; Kahraman et al., 2006), a simplified bifurcation geometry of an iliac artery is built, as shown in Fig. 1. The bifurcation is modelled so that the cross section at the end of the aorta is gradually changed from a circle to an ellipse. This is smoothly connected to the two iliac arteries via cubic spline positional polylines using Matlab (The MathWorks Inc., Natick, USA). We model the iliac bifurcation as a two-layer thick-walled structure, and the thickness ratio between the medial and adventitial layers is taken to be 4:3 (Schriefl et al., 2012). A total of seven hexahedron elements through the wall thickness is constructed, with four in the media (shown in red in Fig. 1) and three in the adventitia. Although the geometry is symmetric and the modelling could be achieved by considering a quarter of the whole section, we choose to use the whole geometry so that the modelling can be easily extended to include the fluid–structure interaction in future studies, for which the flow field can be asymmetric. We also note that the 3D simulation of the whole section is fast (within minutes on a Dell workstation with 6 CPU cores, 2.9 GHz and 32 G memory).

### 2.2. Constitutive model

The model of Holzapfel et al. (2000) assumes that the strain–energy function *Ψ* is the sum of an isotropic potential *Ψ*_iso_ associated with the ground matrix and an anisotropic potential *Ψ*_aniso_ associated with the embedded families of perfectly aligned collagen fibres (Holzapfel and Weizsäcker, 1998). Assuming that the artery material is incompressible, we have the local volume ratio *J* = det **F** = 1, where **F** is the deformation gradient relative to the unloaded configuration. We also assume that the two fibre families are aligned in the directions of the unit vectors **a**_01_ and **a**_02_ in the unloaded configuration. These are symmetric and lie in the tangent plane (no radial component). Let *λ_r_*, *λ_θ_* and *λ_z_* be the principal stretches in the cylindrical system. Then, the strain–energy function^1^ associated with the right Cauchy–Green tensor **C** = **F**^T^**F** is

(1) $$\begin{array}{l} \Psi =\Psi_{\text{iso}}(C)+\Psi_{\text{aniso}}(C,a_{01},a_{02})\\ =\frac{c}{2}(I_{1}-3)+\frac{k_{1}}{2k_{2}}\sum_{i=4,6}\left[\exp(k_{2}{\left(I_{i}-1\right)}^{2})-1\right], \end{array}$$

with the *I*_4_ term only included if *I*_4_>1 and the *I*_6_ term only included if *I*_6_>1. The constants *c*>0, *k*_1_>0 are stress-like material parameters and *k*_2_>0 is a dimensionless material parameter. The invariants are *I*_1_ = tr **C**, *I*_4_ = **a**_01_ · (**Ca**_01_), and *I*_6_ = **a**_02_ · (**Ca**_02_), where *I*_4_ and *I*_6_ represent the squared stretches in the fibre directions **a**_01_ and **a**_02_ respectively. For the walls of most large arteries, these two fibre families are located symmetrically about the axial direction, so that, for the deformation considered here, $I_{4}=I_{6}=\lambda_{\theta }^{2}\cos^{2}\beta +\lambda_{z}^{2}\sin^{2}\beta$, where *β* denotes the angle between **a**_01_ (or **a**_02_) and the circumferential direction of the artery.

The *κ* model extends the model of Holzapfel et al. (2000) by changing the anisotropic part to (Gasser et al.,2006)

(2) $$\Psi_{\text{aniso}}=\frac{k_{1}}{2k_{2}}\sum_{i=,4,6}\left[\exp(k_{2}{\hat{E}}_{i}^{2})-1\right],$$

where

$${\hat{E}}_{i}=\kappa I_{1}+(1-3\kappa )I_{i}-1,\;\;\;i=4,6,$$

and *κ* ∈ [0, 1/3] is a dispersion parameter (the same for each fibre family). Notice that when *κ* = 0, the *κ* model is the same as the one published in Holzapfel et al. (2000), and when *κ* = 1/3 we recover an isotropic potential similar to that used in Demiray (1972).

The Cauchy stress tensor is given by

(3) $$\sigma =-pI+2F\frac{\partial \Psi }{\partial C}F^{T},$$

where *p* is a *Lagrange multiplier*, and **I** is the identity tensor, and for the considered model this is given by

(4) $$\sigma =-pI+cb+2k_{1}\sum_{i=4,6}{\hat{E}}_{i}\exp\;\left(k_{2}{\hat{E}}_{i}^{2}\right)[\kappa b+(1-3\kappa )(a_{i}⊗a_{i})],$$

in which **b** = **FF**^T^ is the left Cauchy–Green tensor and **a***_i_* = **Fa**_0_*_i_*, *i* = 1, 2.

**Fig. 2:**
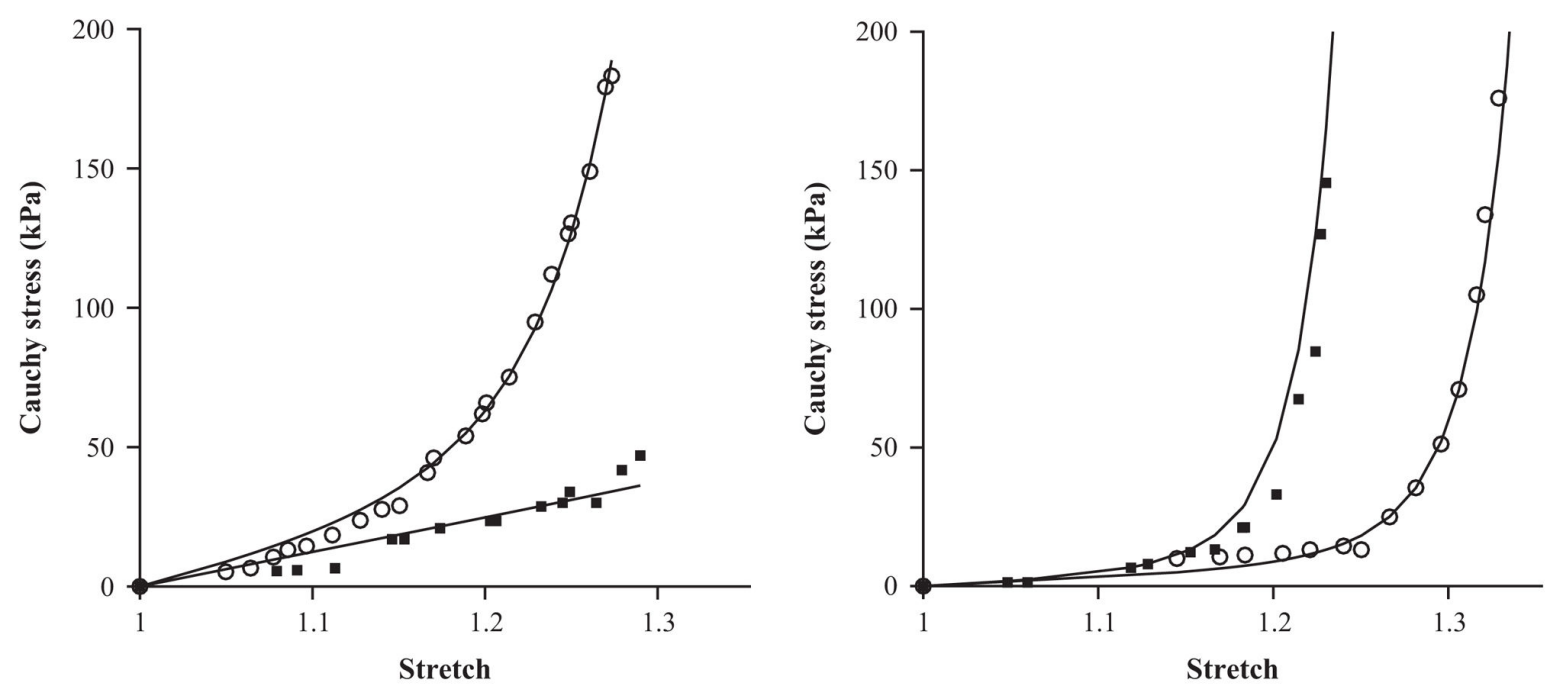
The circumferential and axial stress–stretch responses of the medial (left) and the adventitial (right) layers of a human iliac artery fitted to experimental data. The experimental data shown in symbols (circumferential: circle; axial: square) are from sample no. IV in Holzapfel et al. (2004). The parameters used are listed in Table 1.

The fitting procedure is the standard Levenberg–Marquardt algorithm (Moré, 1978) and it is realized using the Matlab function *lsqnonlin*. The parameters obtained are found to be unique when we select suitable upper and lower bands for the searching ranges in the algorithm (e.g., *c*_0_, *k*_1_, *k*_2_ ∈ [0, 10 000], *κ* ∈ [0, 1/3]). The fitted material and structural parameters of a representative human iliac artery referring to the medial and adventitial layers are given in Table 1, with the azimuthal angles *β^j^*, *j* = M, A determined from biaxial experiments (Schriefl et al., 2012). We assume that the material properties of the abdominal aorta are the same as for the descending common iliac arteries. Fig. 2 shows a comparison between the experimental data and the fitted results obtained from the *κ* model in Eq. (2) for both the circumferential and axial directions of the medial and adventitial layers in the iliac artery. The ‘goodness of fit’, as defined in Schulze-Bauer et al. (2003) and Schriefl et al. (2012), is 0.09 and 0.16, respectively. Note that the axial stress–strain curve in the medial layer is not significantly exponential (it appears to be almost linear). This is presumably because the fibres are more aligned towards the circumferential direction, hence do not contribute much to the exponential term in the axial direction, particularly when the stretch is smaller than 1.25.

### 2.3. Finite element simulation

The finite element simulations are performed using the commercially available finite element package (ABAQUS, 2013). For a typical simulation we use a total of 54 096 hexahedron elements (C3D8H: linear elements using a hybrid formulation) for a bifurcation model, with 19 152 elements for the aorta, and 17 472 for each branch of the common iliac artery. The grid size is chosen following a grid independence test (simulations were run for increasingly refined grids until the results converged). Each branch of the bifurcation is subjected to an in vivo axial pre-stretch of *λ_z_* = 1.07 (Schulze-Bauer et al., 2003), defined as the ratio of in situ length to ex situ length (Schulze-Bauer et al., 2003; Holzapfel et al., 2007), and a transmural physiological mean pressure of 100 mmHg (Holzapfel et al., 2000; Schulze-Bauer et al., 2003; Ohayon et al., 2005). The circumferential displacements of the inlet and outlets are fixed by setting these to zero in the locally cylindrical coordinate systems.

The simulation was run with different alignments of the fibres in the media of the descending iliac artery between *β*^M^ = 0° and 50°. The upper limit of 50° is used as it is widely accepted that the medial layer tends to support more circumferential than axial stresses, because the fibre orientation is closer to the circumferential direction than the axial direction (Schriefl et al., 2012). When *β*^M^ = 0°, the two families of fibres merge to a single family aligned in the circumferential direction. Except in the energy optimisation method discussed below, in all other simulations the fibre angle *β*^A^ of the adventitia is fixed at 53.8°, following measurements documented in Schriefl et al. (2012).

## 3. Criteria to determine the optimal angle

In order to determine the optimal fibre angle, we consider three different hypotheses that may explain the optimal fibre orientation in iliac arteries. These are based on the uniformity factor of the transmural stress distribution, stress-driven remodelling, and energy arguments.

### 3.1. Hypothesis I: uniformity factor

This approach assumes that the fibres are aligned so that, under the mean pressure and an axial pre-stretch, the transmural gradient of the maximum principal Cauchy stress *σ_θ_* is minimised. This is a hypothesis initially proposed by Fung (1983) based on experimental observations of arteries. The advantage of a uniform stress in physiological terms is that cells within tissues are in a homeostatic state of stress, maintained by the biological remodelling process (Artmann and Chien, 2008). To start with, we assume that the unloaded configuration is stress-free (but this assumption is discarded in Section 3.1.2). In accordance with Holzapfel and Gasser (2007), the distributions of these stresses are considered across the deformed wall thickness (including the medial and the adventitial layers). To quantify the uniformity of the circumferential stress throughout the artery wall, we adopt the definition of the standard deviation as the uniformity factor (UF) (Delfino et al., 1997), i.e.

(5) $$\text{UF}={\left(\frac{1}{N-1}\sum_{n=1}^{N}{\left(\sigma_{\theta n}-{\bar{\sigma }}_{\theta }\right)}^{2}\right)}^{1/2},$$

where *σ_θn_* is the maximum circumferential Cauchy stress of the *n*th sampling point through the iliac artery wall (the daughter branches) and *σ̄_θ_* is the mean value of *σ_θn_* across the wall. The number of sampling points *N* herein is chosen to be 7.

**Fig. 3:**
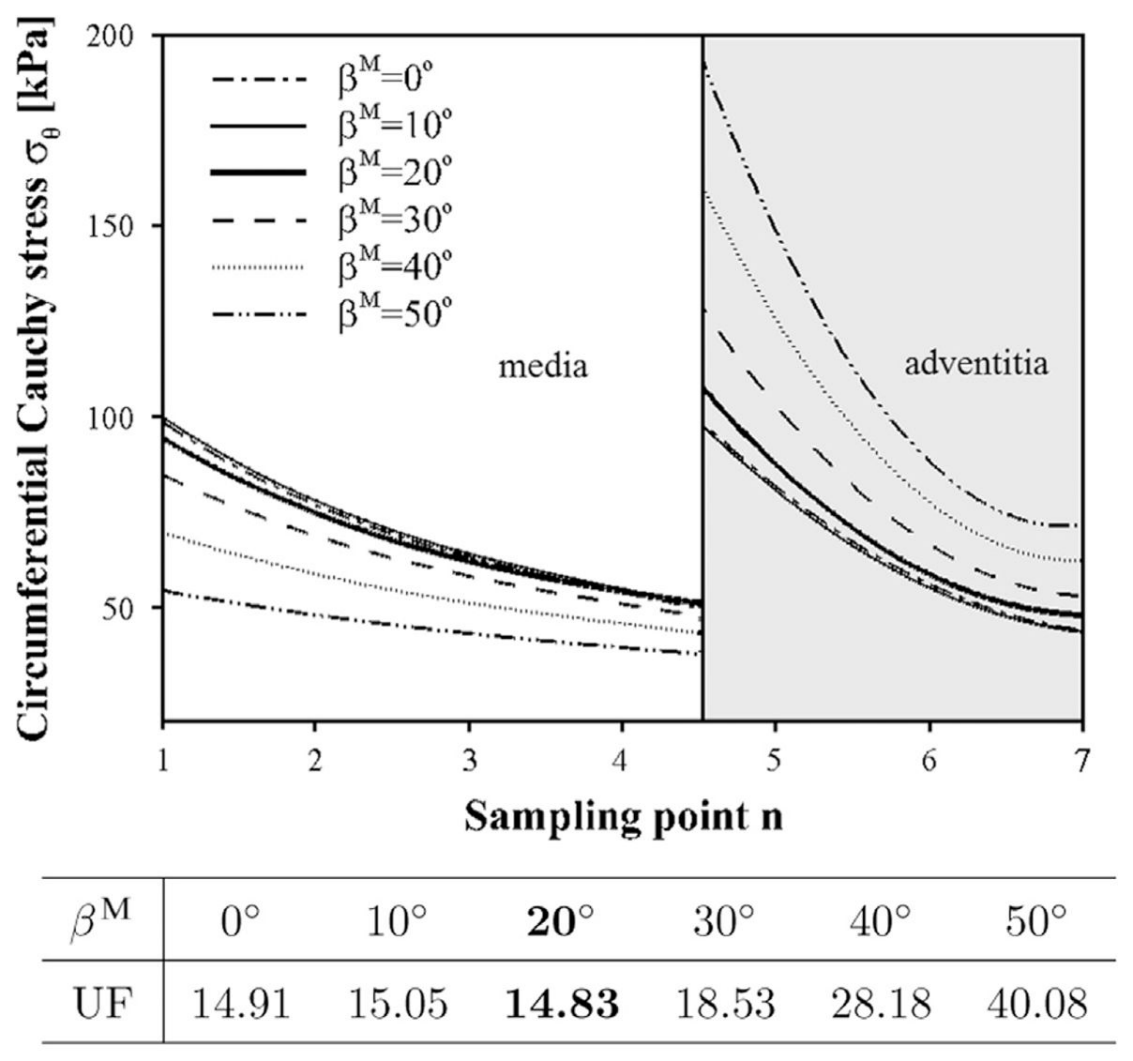
Transmural stress distribution *σ_θ_* at different medial fibre angle alignments. The *x*-axis shows the sampling point *n* from the inner to the outer radius. The curves of the circumferential stress *σ_θ_* are interpolated from the centre points of the finite elements. The thick solid curve at *β*^M^ = 20° is shown to be more uniform than all other angles since its UF value is the smallest, as shown in the table.

The transmural stress distributions at the pre-stretch of 1.07 are plotted in Fig. 3, indicating a distinct jump between the medial and adventitial layers. This agrees with published works (Gao et al., 2006; Holzapfel and Gasser, 2007), and is caused by the fact that different material parameters are used for the medial and adventitial layers. The overall transmural stress distribution across the two layers is the most uniform for *β*^M^ = 20°, though the difference is small compared with that for *β*^M^ = 0°.

#### 3.1.1. Comparison between the bifurcation and the tube structure

To distinguish the effect of the pre-stretch from the bifurcation geometry, the simulations were run for several axial pre-stretches in addition to the physiological value of *λ_z_* = 1.07, for both the bifurcation and the straight tube structure. All other parameters and loading conditions are kept the same as for the tube model. The best (meaning the stress is most uniform at that angle for the number of angles studied) fibre angles from the UF criterion are listed in Table 2.

Interestingly, Table 2 shows that the best fibre angles of the bifurcation and the tube structure are very similar. In other words, the effect of the pre-stretch seems to be much more important than the geometrical effects since, for a given pre-stretch, the best fibre angle is basically the same in either the bifurcation or the tube model under this hypothesis. This important observation suggests that we may now focus on the effects of the pre-stretch using the tube model for which the analytical solutions can be easily derived.

#### 3.1.2. Effect of the circumferential residual stress

Since Table 2 shows that the optimal fibre angle is 20°, and not 0°, it suggests that our UF model has not captured all the important factors. One possibility is due to the fact that the unloaded configuration is not stress free as we have assumed in our previous calculation. Several studies have shown that the circumferential residual stress can change the stress distribution through the thickness (Chuong et al., 1986; Takamizawa and Hayashi, 1987; Delfino et al., 1997; Greenwald et al., 1997; Auricchio et al., 2014).

**Fig. 4:**
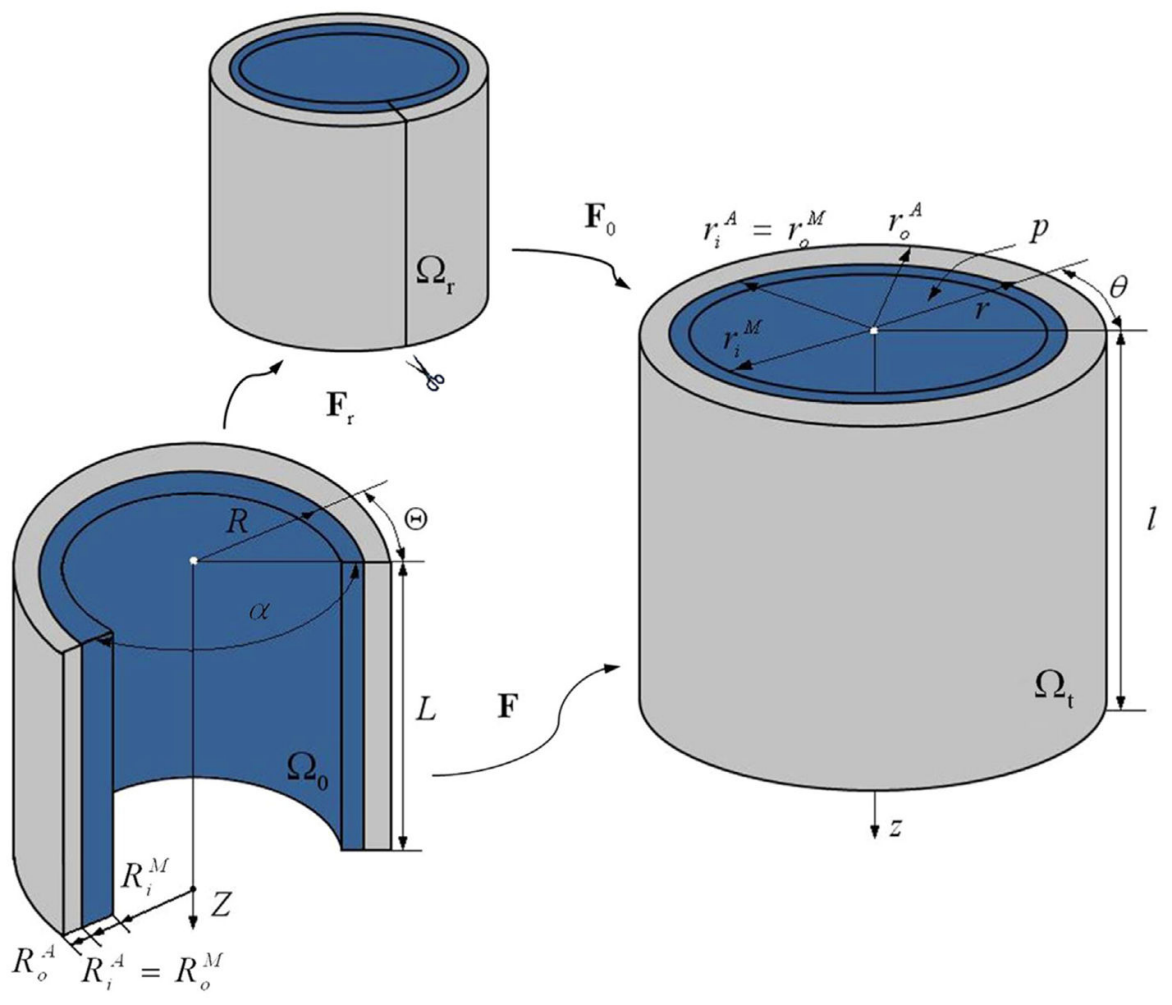
Cylindrical artery wall in the stress-free configuration *Ω_r_*, the unloaded configuration *Ω*_0_, and the current configuration *Ω_t_*, replotted following Waffenschmidt and Menzel (2014).

To address this issue we introduce a circumferential residual stress based on the opening angle method (Chuong and Fung, 1986). Let *α* denote the opening angle in the reference configuration, as depicted in Fig. 4. Then, in terms of cylindrical polar coordinates (*R*, *Θ*, *Z*), the geometry of the tube is defined by

(6) $$R_{i}^{M}\leq R\leq R_{o}^{A},\;\;\;0\leq \Theta \leq 2\pi -\alpha ,\;\;\;0\leq Z\leq L,$$

where $R_{i}^{M}$ and $R_{o}^{A}$ denote the inner radius of the medial layer and the outer radius of the adventitial layer, respectively, while *L* is the length of the undeformed sector. For continuity, we also have $R_{o}^{M}=R_{i}^{A}$. Note that the opening angle identified in Fig. 4 differs from the definition used in Fung and Liu (1989) and Zulliger et al. (2004).

In terms of coordinates (*r*, *θ*, *z*), the geometry of the current configuration is given by

(7) $$r_{i}^{M}\leq r\leq r_{o}^{A},\;\;\;0\leq \theta \leq 2\pi ,\;\;\;0\leq z\leq l,$$

where $r_{i}^{M},r_{o}^{A}$ and *l* denote the inner and the outer radius and the length of the deformed tube, respectively, with $r_{o}^{M}=r_{i}^{A}$.

The deformation gradient **F** is then the composition of the deformation gradient **F**_0_ relative to the unloaded configuration, and **F***_r_* relative to the stress-free configuration, as indicated in Fig. 4. Thus,

(8) $$F=F_{0}F_{r}.$$

Using the cylindrical coordinates we have **x** = *r***e***_r_* + *z***e***_z_*, where (**e***_r_*, **e***_θ_*, **e***_z_*) are the unit basis vectors in the current configuration. For our problem

(9) $$r=\sqrt{\frac{R^{2}-{\left(R_{i}^{M}\right)}^{2}}{k\lambda_{z}}+{\left(r_{i}^{M}\right)}^{2}},\;\;\;\theta =k\Theta ,\;\;\;z=\lambda_{z}Z,$$

where *λ_z_* is the (constant) axial pre-stretch, and *k* = 2*π*/(2*π* − *α*).

By incompressibility, *λ_r_λ_θ_λ_z_* = 1. Hence, we have

(10) $$\lambda_{r}(R)=\frac{R}{rk\lambda_{z}},\;\;\;\lambda_{\theta }(R)={\left(\lambda_{r}\lambda_{z}\right)}^{-1}=\frac{rk}{R}.$$

The deformation gradient is then

(11) $$F=\lambda_{r}e_{r}⊗E_{R}+\lambda_{\theta }e_{\theta }⊗E_{\Theta }+\lambda_{z}e_{z}⊗E_{Z},$$

with *λ_m_*, *m* = *r*, *θ*, *z* being the principal stretches in the radial, circumferential and axial directions, respectively, and **E***_m_*, *m* = *R*, *Θ*, *Z*, are the unit basis vectors in the reference configuration. In the absence of body forces and by assuming no external pressure, the internal pressure *P* is

(12) $$P=\int_{r_{i}^{M}}^{r_{o}^{A}}(\sigma_{\theta }-\sigma_{r})\frac{dr}{r},$$

where *σ_θ_* and *σ_r_* are the principal Cauchy stresses in the circumferential and the radial directions, respectively.

We need to make one assumption on the kinematics in order to make progress. To be specific, we assume that the wall thickness does not change between the initial and the stress-free configuration, following the studies of Delfino et al. (1997) and Holzapfel et al. (2000), and by making use of the incompressibility condition, we obtain

(13) $$k(R_{o}^{2}-R_{i}^{2})=r_{o}^{2}-r_{i}^{2},$$

where *R*_o_ = *R*_i_ + *H* and *r*_o_ = *r*_i_ + *H*, and *H* is the wall thickness. This allows us to solve Eq. (12) numerically using a Gaussian integration scheme (Holzapfel et al., 2000). The geometrical parameters used in the simulations are summarised in Table 3.

Table 4 illustrates that the minimum value of UF is reached when the optimal fibre angle is around 0° at the pre-stretch 1.07 after including the circumferential stress. This now agrees with the experiments. It also shows the minimum value of UF at different values of *λ_z_*, which indicates a strong dependence of optimal *β*^M^ on *λ_z_*. It seems that the medial fibres tend to be aligned in the circumferential direction when the pre-stretch is below 1.14. However, *β*^M^ increases sharply (>30°) for *λ_z_*>1.15 and the transmural stress distribution becomes more uneven. This is probably the reason why in human samples the corresponding angular derivation of the mean fibre angle in the media of iliac artery is close to 0° (Schriefl et al., 2012).

### 3.2. Hypothesis II: stress-driven remodelling

**Fig. 5:**
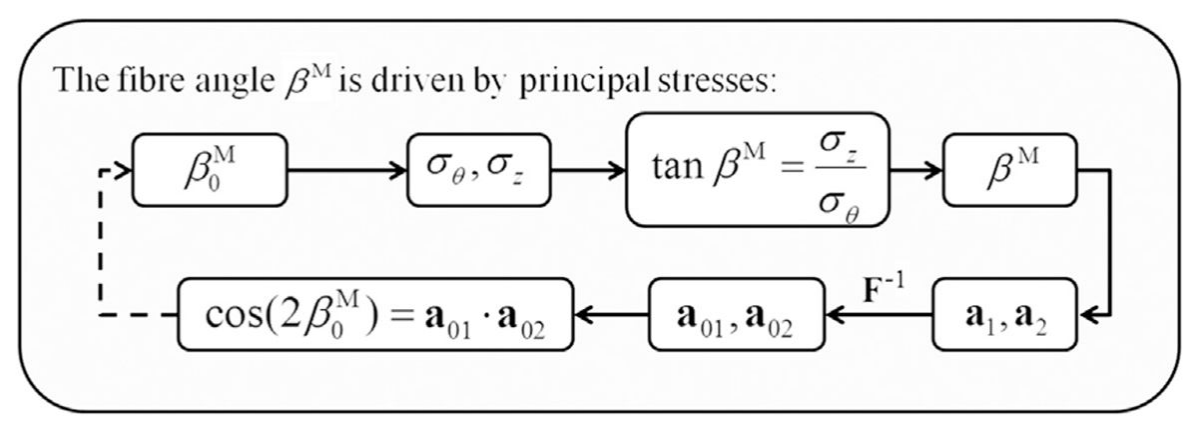
Inverse procedure for determining the fibre orientation using the stress-driven criterion. This process converges to the solution for any values of $\beta_{0}^{M}$ between 0° and 50°.

This hypothesis assumes that the fibres adapt during the remodelling process so that the artery layers have optimal load-bearing capability. Here we adopt a simple stress-driven remodelling model proposed by Hariton et al. (2007), which assumes that the two families of collagen fibres are aligned between the principal stretch directions as dictated by the ratio of the magnitudes of the two largest principal stresses *σ_θ_* and *σ_z_* (Driessen et al., 2004; Hariton et al., 2007). In the present study, only the collagen fibre orientation in the medial layer is adjusted due to the remodelling process. Since remodelling requires the solution of an inverse problem, an iterative finite element based procedure is developed, as shown in Fig. 5.

The Cauchy stress is given by

$$\sigma =\sigma_{\theta }e_{\theta }⊗e_{\theta }+\sigma_{z}e_{z}⊗e_{z}+\sigma_{r}e_{r}⊗e_{r},$$

where *σ_m_* (*m* = *r*, *θ*, *z*) are the principal Cauchy stresses and **e***_m_* (*m* = *r*, *θ*, *z*) are the principal directions. Following Driessen et al. (2004) and Hariton et al. (2007), we assume that the angle *β*^M^ of alignment between the fibre direction obeys

(14) $$\tan\beta^{M}=\frac{\sigma_{z}}{\sigma_{\theta }},$$

whereby the fibres are assumed to be in the plane spanned by the vectors aligned with the two largest principal stresses, and the collagen fibres are symmetrically aligned relative to **e***_θ_*, the direction of the maximal principal stress. The unit vectors along the two families of collagen fibres are, in the current configuration,

$$a_{i}=\cos\beta^{M}e_{\theta }\pm \sin\beta^{M}e_{z},$$

and in the reference configuration,

$$a_{0i}=\frac{F^{-1}a_{i}}{|F^{-1}a_{i}|},\;\;\;i=1,2.$$

The updated fibre alignment in the reference configuration is then calculated from

$$\cos(2\beta_{0}^{M})=a_{01}\cdot a_{02}.$$

The remodelling procedure terminates when the maximal absolute variance of the mean fibre orientation between the current and last steps converges to a set tolerance, i.e. smaller than 0.01°. For each iteration, we assume the artery is in a quasi-static condition.^2^

**Fig. 6:**
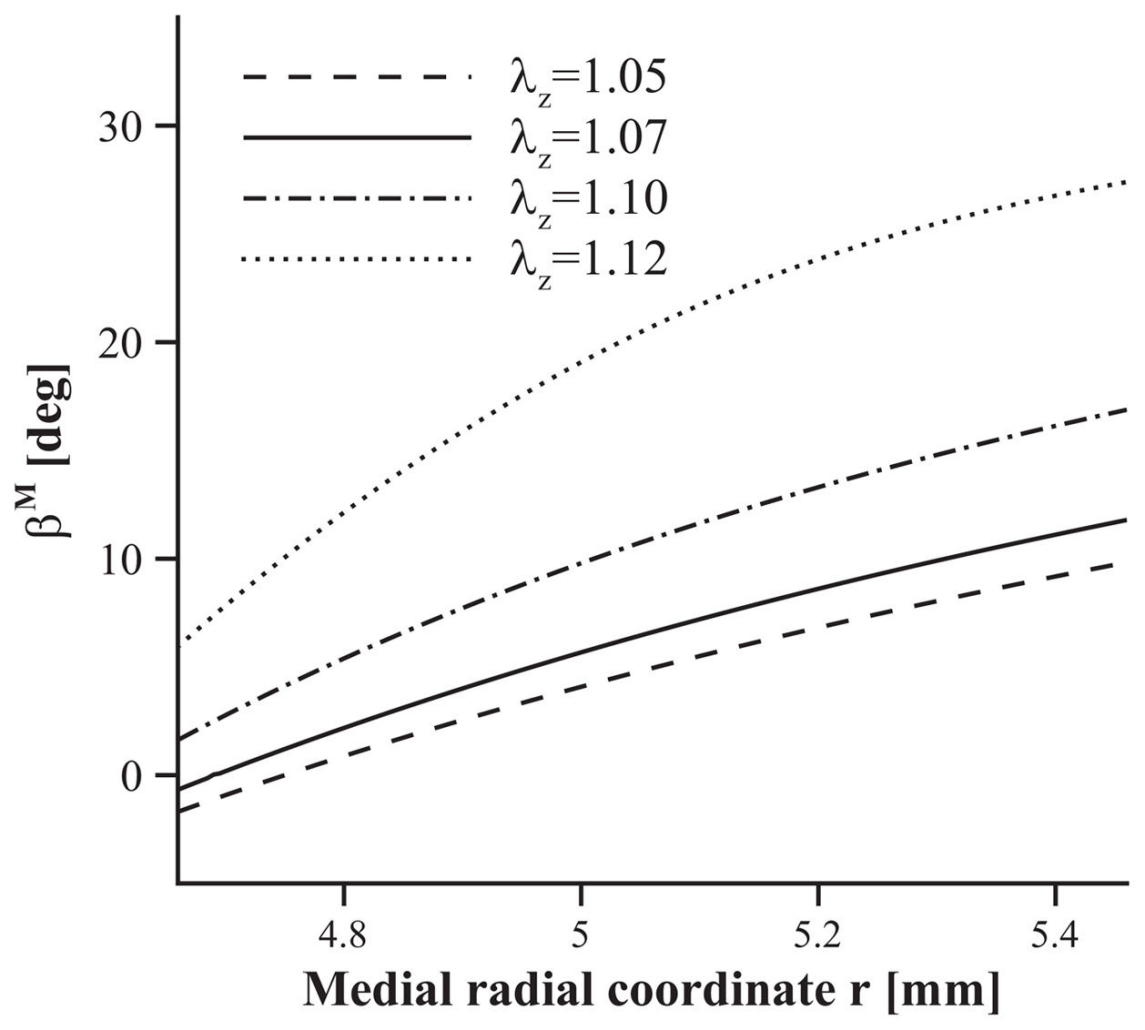
Dependence of the remodelled fibre orientation (characterized by *β*^M^) on the medial radial coordinate *r* as a function of the axial pre-stretch given as 1.05 (dashed), 1.07 (solid), 1.10 (dash-dotted) and 1.12 (dotted). Residual strains are included (*α* = 160 deg).

Fig. 6 provides the results of the remodelling process with the circumferential residual stress. The mean fibre orientation across the artery wall of the medial layer is 6.3° at a pre-stretch of *λ_z_* = 1.07. The result is reasonably close to zero degrees and suggests that the fibres are mostly circumferentially oriented. The influence of the axial pre-stretch is also shown in Fig. 6. On the whole, the values of the fibre angle increase with *λ_z_*, while the range of *β*^M^ becomes wider when *λ_z_* becomes larger. Note that if *λ_z_* is assigned an even larger number (for example, *λ_z_* = 1.13), the iterative system becomes unstable, and it is difficult to find converged solutions. This is because the fibre angle update is determined by the ratio between the maximal and the second principal stresses. When the pre-stretch is greater than 1.13, the maximal principal stress changes from the circumferential stress to axial stress. At the transition phase, the iteration is oscillating between those two stresses. It would appear that for *λ_z_*>1.13, there should be a change in the remodelling criter-ion. However, since the physiological value of *λ_z_* is less than 1.13, we do not pursue this further. The negative fibre angles proximal to the inner radius are due to the flip-over of the two fibre directions. Though uniformly stretched, the inner wall in the axial direction is under compression, in other words, the medial principal stresses *σ_z_*<0, and consequently, the *β*^M^ given by Eq. (14) changes its sign. The marginal differences in the optimal angles compared with the UF criterion may be due to the oversimplified criterion of the remodelling model used.

### 3.3. Hypothesis III: energy minimisation

We now determine the optimal fibre orientation based on the energy argument recently proposed by Waffenschmidt and Menzel (2014), which assumes that the fibres are aligned so that the minimum of total potential energy *Π* is maximised with respect to *β*^M^ and *β*^A^. For a hyperelastic material, *Π* is the sum of the elastic strain energy *Π*_int_ stored in the deformed body and the potential energy *Π*_ext_ of the applied forces, expressed as *Π* = *Π*_int_ + *Π*_ext_ + const. The main objective is to access information on preferred material, structural and loading parameters that are associated with the extremal states of the total energy, and to use these to identify the favourable configurations for the design and adaptation of artery walls. Specifically, the total energy for a tube model can then be expressed as

(15) $$\Pi =2\pi l\int_{r=r_{i}^{M}}^{r_{o}^{A}}\Psi (\lambda_{r},\lambda_{\theta },\lambda_{z})rdr-P\pi {\left(r_{i}^{M}\right)}^{2}l+\text{const}.$$

Note that *λ_z_* is prescribed, and with the incompressibility condition *λ_r_λ_θ_λ_z_* = 1 the strain energy *Ψ* is a function of *β*^M^, *β*^A^, *λ_θ_*_i_ and *α*, where *λ_θ_*_i_ is the inner circumferential stretch.

Since the equilibrium of the system also requires the minimisation of the total strain energy in terms of displacements, the optimisation of the total energy is the result of maximising all the permissible minimised total energies. The solution of the underlying boundary-value problem is obtained by the optimisation of *Π*. The deformation variables (*λ_θ_*_i_, *α*) firstly minimise the total energy, which results in the triple ( $\lambda_{\theta i}^{\min}$, *α*^min^, *Π*^min^). Subsequently, a set of values of *Π*^min^ corresponding to the states of equilibrium for which *Π*^min^ is maximised to render the optimal material parameters ( $\beta_{\text{opt}}^{M},\beta_{\text{opt}}^{A}$), i.e.

(16) $$\{\beta_{\text{opt}}^{M},\beta_{\text{opt}}^{A}\}=\arg\max_{\beta^{M},\beta^{A}}\;\{\min_{\lambda_{\theta i},\alpha }\;\Pi (\lambda_{\theta i},\alpha ,\beta^{M},\beta^{A})\}.$$

The reader is referred to Waffenschmidt and Menzel (2014) for the detailed algorithm that determines the functional *Π* in Eq. (15). The physical interpretation of Eq. (16) suggests that in an arterial tissue the fibres adapt to be aligned so that the tissue’s loading capacity is maximised. Thus, the internal energy is maximised among the minimised values of *Π*, in the case of Dirichlet boundary conditions.

**Fig. 7:**
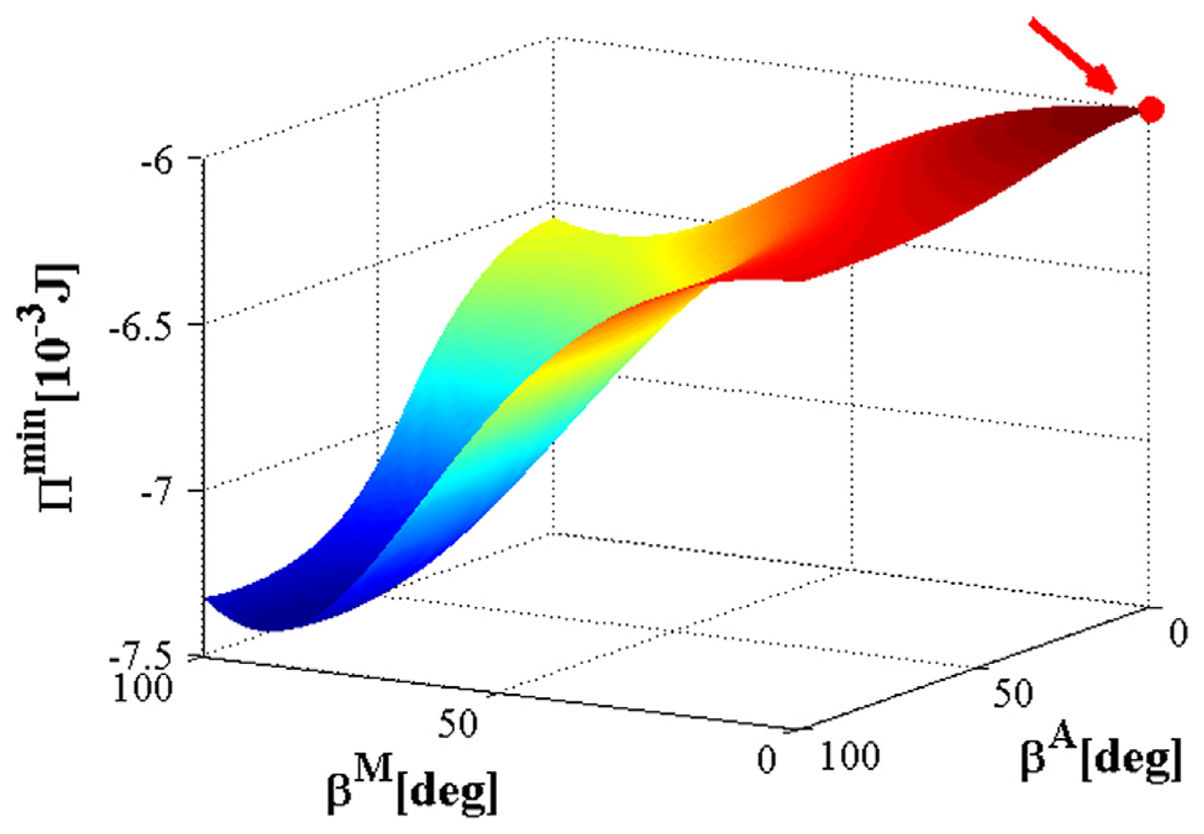
Values of *Π*^min^ plotted against *β*^A^ and *β*^M^. The red dot indicates the maximum value of *Π*^min^. (For interpretation of the references to colour in this figure caption, the reader is referred to the web version of this paper.)

Fig. 7 shows the variation of the total potential energy *Π* in the parameter space of *β*^M^ and *β*^A^. It is evaluated numerically at the physiological pressure of 13.33 kPa and the axial stretch of 1.07. In Fig. 7 the maximum value of *Π*^min^ is indicated by a red dot, which occurs at *β*^M^ = 0°. This is consistent to the results from the previous two hypotheses. The value of *β*^A^ is discussed in the next section.

**Fig. 8:**
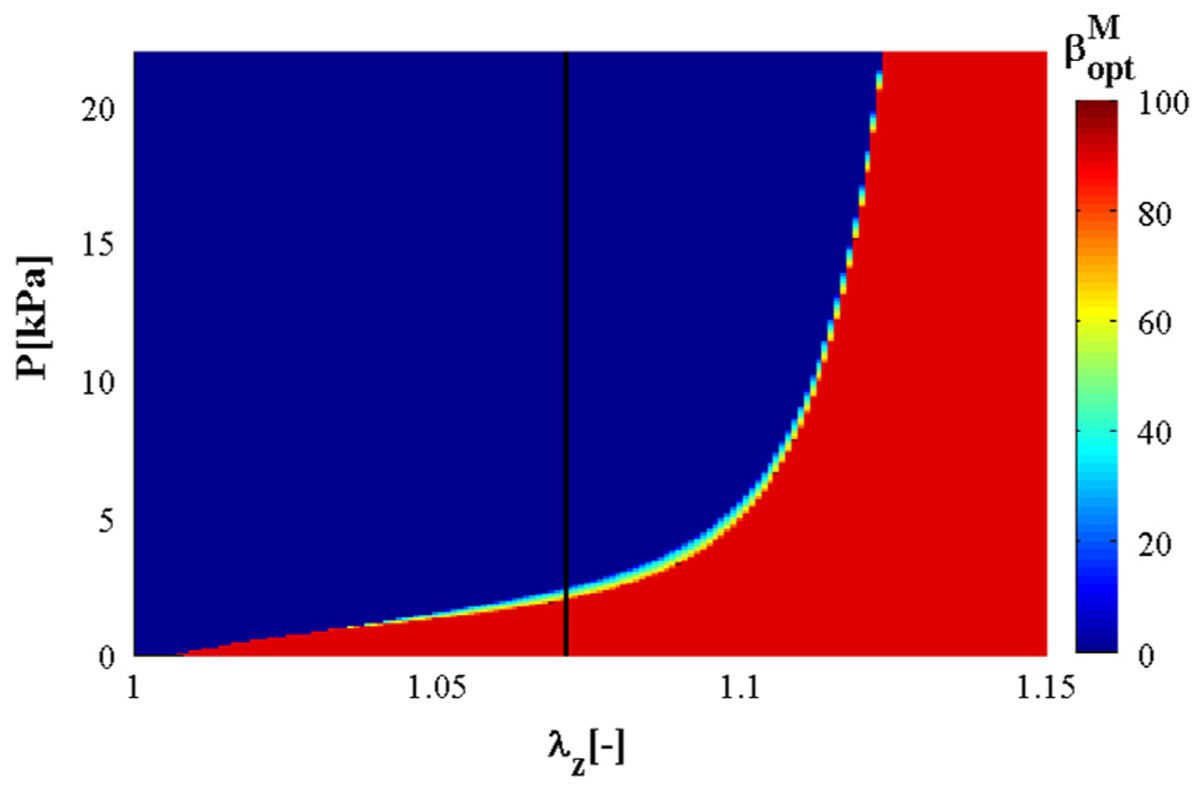
Relation between the axial stretch *λ_z_*, internal pressure *P*, and optimal medial fibre angle $\beta_{\text{opt}}^{M}$, as indicated by the colour bar. The black solid line highlights the variation of $\beta_{\text{opt}}^{M}$ with *P* at *λ_z_* = 1.07; for a wide range of pressure ( $\beta_{\text{opt}}^{M}=0{}^{\circ}$). (For interpretation of the references to colour in this figure caption, the reader is referred to the web version of this paper.)

Following Waffenschmidt and Menzel (2014), we also plot the relation between the axial stretch *λ_z_*, the internal pressure *P* and the optimal medial fibre angle $\beta_{\text{opt}}^{M}$ in Fig. 8, with the colour bar referring to the optimal value of $\beta_{\text{opt}}^{M}$. It is clear that in a wide range of physiological pressure, the optimal fibre angle is oriented towards the circumferential direction, i.e. $\beta_{\text{opt}}^{M}=0{}^{\circ}$. However, if *λ_z_* >1.12, then there is a sudden change of fibre alignment from the circumferential to the axial direction, i.e. $\beta_{\text{opt}}^{M}=90{}^{\circ}$, irrespective of the pressure magnitude.

**Fig. 9:**
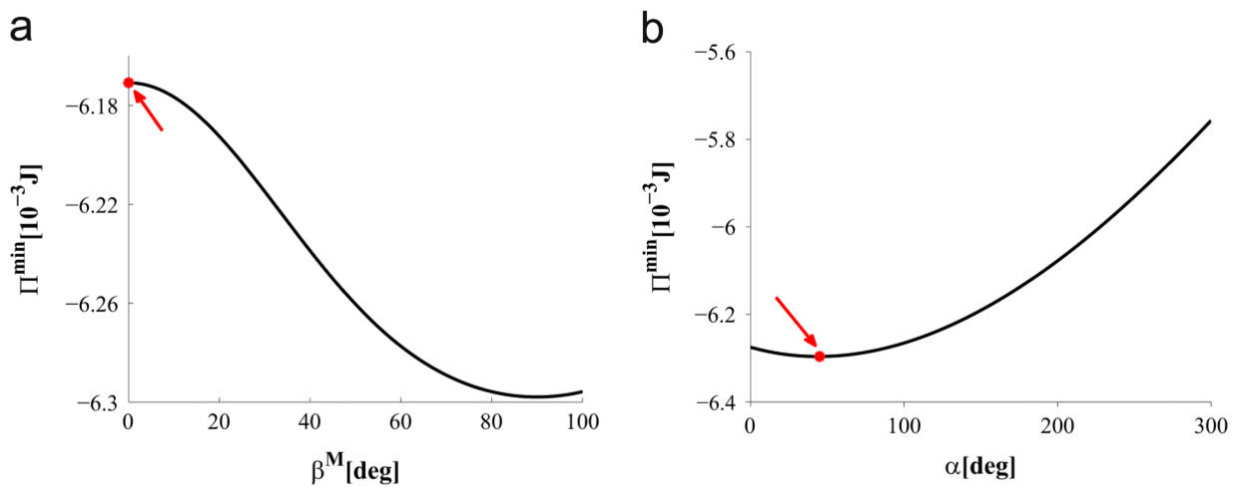
(a) *Π*^min^ versus *β*^M^, with the red dot denoting $\beta_{\text{opt}}^{M}=0\;\deg$ when *Π*^min^ reaches its maximum; (b) *Π*^min^ versus *α*, with the red dot denoting the optimal position at *α* = 45 deg when *Π* is minimised. (For interpretation of the references to colour in this figure caption, the reader is referred to the web version of this paper.)

If we assign *β*^A^ = 53.8° (Schriefl et al., 2012), then the results are similar to the previous two hypotheses, as shown in Fig. 9(a). In particular, we have $\beta_{\text{opt}}^{M}=0{}^{\circ}$, *λ_θ_*_i_ =1.23, and *α* = 45°.

## 4. Discussion

The main result of this paper is to show that by using each of the three different hypotheses there seems to be an optimal mean fibre angle in the media of the human iliac artery in the circumferential direction, as observed in recent experiments of Schriefl et al. (2012). Since we only performed a static analysis, the agreement with experiments seems to suggest that the fibre alignments are dominantly influenced by the static mean physiological loading. In order to accurately estimate the fibre angle we need to include the residual stress effect in the UF approach since the arteries do not recover to the zero-stress configuration when unloaded.

In the first two approaches the fibre orientation of the adventitia is fixed. However, with the energy optimisation method, all parameters can be estimated including the opening angle *α* and the adventitial mean fibre orientation *β*^A^. In all simulations, the importance of the pre-stretch in the determination of the fibre orientation is found to be paramount. This and several other issues are discussed in more detail below.

### 4.1. Role of pre-stretch

Our simulations suggest that the iliac artery has only one family of fibres in the media with preferred circumferential direction. Hence, it is useful to ask what is so special about the iliac artery when compared to the aorta where two families of fibres are always present in each layer? The main explanation, as suggested by our results, comes from the significance of the pre-stretch. In particular, the typical pre-stretch in an iliac artery is around 1.07, yet in most of the large arteries such as the aorta and the carotid artery the pre-stretch is normally larger than 1.1 (Han and Fung, 1995; Holzapfel et al., 2000, 2007; Gasser et al., 2002; Holzapfel and Gasser, 2007; Cardamone et al., 2009).

Indeed, when we apply the UF criterion to rabbit carotid arteries using the data from Holzapfel et al. (2000), with the physiological value of pre-stretch 1.6, we find the optimal medial fibre angle of 31° (see UFs in Table 5), which is close to the average experimental results of 29° (Holzapfel et al., 2000).

Incidentally, the optimal fibre angles of the rabbit carotid artery would also be oriented towards the circumferential direction if the pre-stretches are below 1.45.

We speculate that the reason for the lower value of the pre-stretch of the iliac artery is partially due to the branching structure, though not all bifurcating arteries have sufficiently low pre-stretch to develop a single fibre family. The spatial variation of the pre-stretch along the arterial tree must have been developed optimally through a complex remodelling process under the overall loading conditions, including the dynamic pressure, gravity and fluid–structure interactions, with the interplay of local artery geometries and material properties. For example, in a human carotid bifurcation, the pre-stretch of the layer specific parent branch is very similar to that of the two daughter branches (Sommer et al., 2010). It will be interesting to see more experimental data which may establish a clearer relationship between the pre-stretch and the fibre orientation.

### 4.2. Opening angle in the human iliac artery

**Fig. 10:**
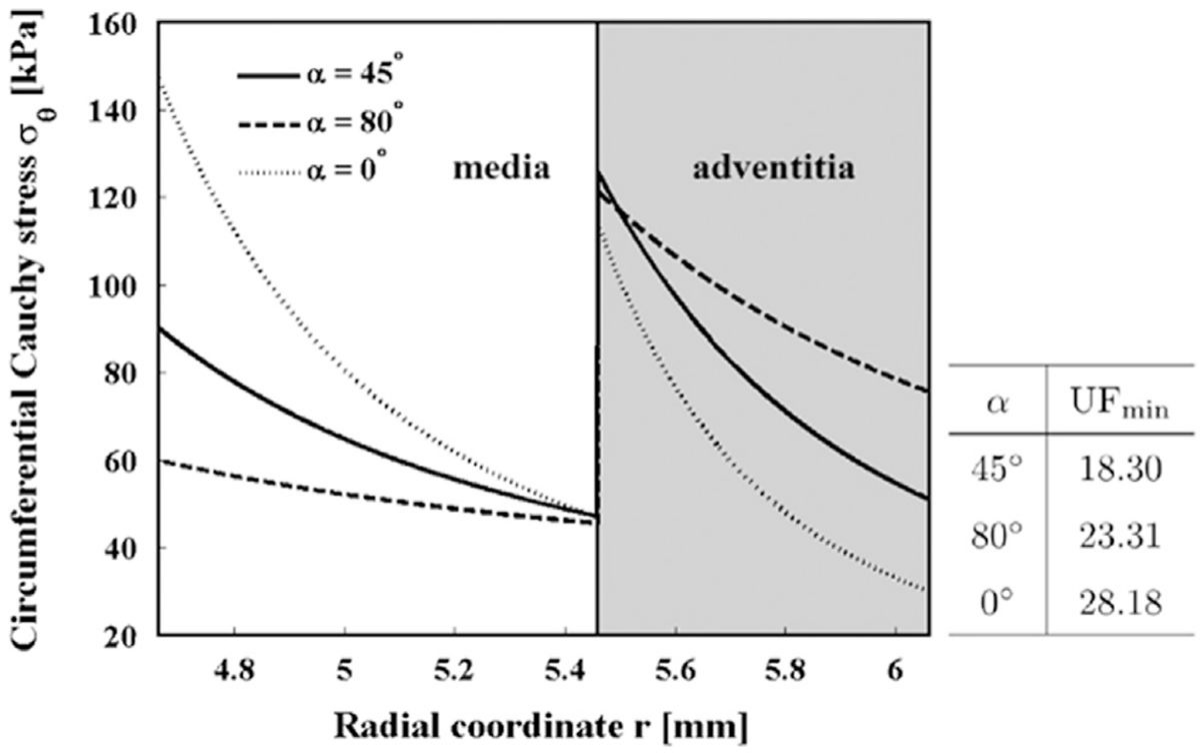
Transmural stress distribution *σ_θ_* at different opening angles. The *x*-axis shows the radial coordinate across the medial and adventitial layers. The stress distribution becomes more uniform when the residual stress (*α* ≠ 0°) is included.

The energy optimisation method suggests that the optimal opening angle *α* is around 45° for a human iliac artery, as shown in Fig. 9(b). This is considerably lower than the published opening angle of 160° found in a rat iliac artery (Fung, 1991; Holzapfel et al., 2000). Since we have no experimental data available to validate this finding, we measured the opening angle in swine iliac arteries of seven healthy adult swines, following the procedure described by Han and Fung (1991) and Han et al. (2006). This measurement confirmed that the opening angle in the swine iliac artery is around 80°. As the swine anatomy bears some similarity to that of the human anatomy, this seems to support our modelling prediction of a smaller human opening angle of 45°. Using the opening angle 45° in the UF approach, we obtained a similar medial fibre angle to that of using 160° (rat), as shown in Table 6. The corresponding stress distributions with and without the opening angles are shown in Fig. 10, which demonstrates that, with the residual stress, the stress distribution is more uniform. This is consistent with the results of Holzapfel et al. (2000).

Indeed, we have used a number of different opening angles and found that the results are unchanged once the opening angle *α* is larger than 20°.

This suggests that although the fibre orientation determined by the UF method requires the residual stress to be included, the final result is not sensitive to the changes of the opening angle as long as the opening angle is larger than a certain value.

Indeed, the zero fibre angle in the medial layer holds true for a wide range of opening angles, which makes sense since the in vivo physiological residual stress must fluctuate due to the complex remodelling processes.

### 4.3. Adventitia fibre orientation β^A^

In most of the simulations we have fixed the fibre angle *β*^A^ in the adventitial layer. However, with the energy optimisation hypothesis, the estimated *β*^A^ is 0°, which disagrees with the experimental measurement of 53.8°. In fact, Fig. 7 shows that the maximum value of *Π*^min^ is insensitive to the variation of *β*^A^; the curve of *Π*^min^ at *β*^M^ = 0 is rather flat for the whole range of *β*^A^. This finding is consistent with the results on carotid arteries (Waffenschmidt and Menzel, 2014), and suggests that the energy optimisation method alone is not sufficient to determine *β*^A^. Besides, the minor influence of *β*^A^ on the determination of the optimal fibre orientation in the medial layer is further confirmed via the sensitivity test under the other two hypotheses.

At this point, it is worth noting that Spencer et al. (1975) considered a circular cylindrical tube of incompressible ideal fibre-reinforced material in which the reinforcement throughout its thickness is directed along two families of helices making angles of ±*β*^A^. Of particular importance, they argued that $\beta^{A}=\sqrt{2}$, so that *β*^A^ = 54.7° in order to avoid the narrow bands of stress concentration near the surfaces at the inner and outer radii. This value is surprisingly similar to the adventitial fibre angle measured in experiments (Holzapfel et al., 2000; Schriefl et al., 2012). However, this should be viewed with caution since the presence of fibres is not exactly equivalent to the inextensible case of Spencer et al. (1975), and hence the agreement on the value of *β*^A^ could be a coincidence.

### 4.4. Limitations

We identified that the *κ* model is able to capture the mechanical response of the iliac artery, as, for example, documented in Holzapfel et al. (2004). The parameters of the constitutive models, especially the structural parameter *κ* as introduced in Gasser et al. (2006), are obtained by means of the Levenberg–Marquardt algorithm. With the development of advanced experimental techniques, this particular parameter can be directly estimated from the measured fibre distribution density by, e.g. a *π*-periodic *von Mises* distribution (Gasser et al., 2006). However, such estimates are often different from the fitted values for the human iliac arteries (Schriefl et al., 2012). An improved description of the mechanics of artery walls at the microscopic level which can incorporate fibre–fibre interactions, fibre recruitment and viscoelasticity (Stylianopoulos and Barocas, 2007; Maceri et al., 2010; Weisbecker et al., 2015) will be required in future in order to make full use of the experimental data.

Other limitations include the fact that we have only performed a static analysis. For the residual stresses, we have used the same constant parameters for the two layers of the artery wall. The stress-free configuration changes over time so that the opening angles for the medial and adventitial layers and an intact artery ring vary significantly (Fung and Liu, 1989; Holzapfel et al., 2005a; Sommer and Holzapfel, 2012). Although the present study reveals the potential link between the fibre orientation and the pre-stretch, we are yet unable to explain the reason for requiring different values of the pre-stretch in different sections of arteries. Therefore, enhanced systematic studies including the dynamic loading, fluid–structure interaction, and possibly tissue remodelling at the cellular level as well as measurements of fibre angles and pre-stretches for other arteries are required.

## 5. Conclusion

From a mechanical point of view, we have suggested an explanation for the rather unusual fibre distribution in the medial layer of the human common iliac artery. Three approaches have been used, namely a uniform distribution of the transmural stress, fibre stress remodelling, and optimisation of the total energy. All three approaches suggest that the optimal fibre angle in the medial layer of human iliac arteries is zero relative to the circumferential direction, as documented in Schriefl et al. (2012). In particular, we have found that the axial pre-stretch is key for explaining the optimal fibre distribution, and the particularly low value in the iliac artery is directly associated with the single fibre family. Moreover, we have shown the necessary involvement of the residual stress when utilising the UF approach, and speculate that the opening angle in human iliac artery is around 45°. Finally, it is likely that the optimal fibre angle in the adventitia is determined by a different optimisation principle to that of the medial layer such as dynamic loading and fluid–structure interaction, which is a topic for a future study.

## Figures and Tables

**Table 1:** Layer-specific material and structural parameters for a human common iliac artery based on the *κ* model.

Parameters	*c* (kPa)	*k*_1_ (kPa)	*k*_2_	*κ*	*β^j^*, *j* = M, A (deg)
Media	20.99	29.34	19.33	0.20	0
Adventitia	8.74	55.09	328.27	0.26	53.8

**Table 2:** Best fibre angles with different pre-stretches for the bifurcation and the tube structure.

*λ_z_*	1.05	1.07	1.08	1.09	1.10	1.12	1.20
Bifurcation structure	30°	**20**°	10°	0°	0°	0°	0°
Tube structure	30°	**20**°	10°	0°	0°	0°	0°–10°

**Table 3:** Geometrical parameters used for the iliac artery, chosen from Schulze-Bauer et al. (2003), Kahraman et al. (2006) and Schriefl et al. (2012). As no measured opening angle is available for human iliac arteries, the corresponding opening angle of rat is used (Fung, 1991; Holzapfel et al., 2000) for both the medial and the adventitial layers.

Parameter	Description	Value	
		Media	Adventitia
*H* (mm)	Wall thickness	0.8	0.6
*R*_i_ (mm)	Inner referential radius	8.9	9.7
*R*_o_ (mm)	Outer referential radius	9.7	10.3
*r*_i_ (mm)	Inner current radius	4.7	5.5
*r*_o_ (mm)	Outer current radius	5.5	6.1
*α* (deg)	Opening angle	160	160

**Table 4:** Minimum value of UF and corresponding *β*^M^ obtained for different values of pre-stretch *λ_z_*, when including the opening angle (*α*=160°).

*λ_z_*	1.00	1.05	1.07	1.10	1.14	1.15	1.17	1.20
*β*^M^	0°	0°	**0**°	0°	0°	30°	41°	57°
UF_min_	12.84	38.00	**48.48**	59.75	69.95	71.80	72.98	73.13

**Table 5:** Optimal medial fibre angle *β*^M^ and corresponding UF_min_ at various values of pre-stretches *λ_z_* for a rabbit carotid artery.

*λ_z_*	1.10	1.40	1.45	1.50	1.55	1.60	1.65	1.70
*β*^M^	0°	0°	0°	2°	13°	31°	42°	50°
UF_min_	49.79	71.86	75.62	79.29	82.65	83.82	81.52	74.92

**Table 6:** Optimal medial fibre angle *β*^M^ at various values of the pre-stretch *λ_z_* for human iliac arteries when the opening angle is set to 45°.

*λ_z_*	1.07	1.10	1.16	1.17	1.18	1.19	1.20
*β*^M^	**0**°	0°	0°	30°	42°	50°	55°
UF_min_	**18.30**	34.89	60.41	62.23	62.73	62.81	62.89

## Footnotes

1 Note, this strain–energy function is designed as an incompressible formulation. A modified version of Eq. (1) with an isochoric/volumetric split is used in the finite element implementation.

2 The term ‘quasi-static’ means that we perform a static analysis because the loading variation with respect to time is considered to be slow.
